# Saposin C promotes survival and prevents apoptosis via PI3K/Akt-dependent pathway in prostate cancer cells

**DOI:** 10.1186/1476-4598-3-31

**Published:** 2004-11-17

**Authors:** Tae-Jin Lee, Oliver Sartor, Ronald B Luftig, Shahriar Koochekpour

**Affiliations:** 1 Department of Microbiology, Immunology, and Parasitology, Louisiana State University Health Sciences Center, 533 Bolivar Street, CSRB 4-17, New Orleans, LA 70112, USA; 2 Stanley S. Scott Cancer Center, Louisiana State University Health Sciences Center, 533 Bolivar Street, CSRB 4-17, New Orleans, LA 70112, USA; 3 Department of Medicine, Louisiana State University Health Sciences Center, 533 Bolivar Street, CSRB 4-17, New Orleans, LA 70112, USA

**Keywords:** Saposin C, Prosaposin, Prostate Cancer, Apoptosis, Survival

## Abstract

**Background:**

In addition to androgens, growth factors are also implicated in the development and neoplastic growth of the prostate gland. Prosaposin is a potent neurotrophic molecule. Homozygous inactivation of *prosaposin *in mice has led to the development of a number of abnormalities in the male reproductive system, including atrophy of the prostate gland and inactivation of mitogen-activated protein kinase (MAPK) and Akt in prostate epithelial cells.

We have recently reported that prosaposin is expressed at a higher level by androgen-independent (AI) prostate cancer cells as compared to androgen-sensitive prostate cancer cells or normal prostate epithelial and stromal cells. In addition, we have demonstrated that a synthetic peptide (prosaptide TX14A), derived from the trophic sequence of the saposin C domain of prosaposin, stimulated cell proliferation, migration and invasion and activated the MAPK signaling pathway in prostate cancer cells. The biological significances of saposin C and prosaposin in prostate cancer are not known.

**Results:**

Here, we report that saposin C, in a cell type-specific and dose-dependent manner, acts as a survival factor, activates the Akt-signaling pathway, down-modulates caspase-3, -7, and -9 expression and/or activity, and decreases the cleaved nuclear substrate of caspase-3 in prostate cancer cells under serum-starvation stress. In addition, prosaptide TX14A, saposin C, or prosaposin decreased the growth-inhibitory effect, caspase-3/7 activity, and apoptotic cell death induced by etoposide. We also discovered that saposin C activates the p42/44 MAP kinase pathway in a pertussis toxin-sensitive and phosphatidylinositol 3-kinase (PI3K) /Akt-dependent manner in prostate cancer cells. Our data also show that the anti-apoptotic activity of saposin C is at least partially mediated via PI3K/Akt signaling pathway.

**Conclusion:**

We postulate that as a mitogenic, survival, and anti-apoptotic factor for prostate cancer cells, saposin C or prosaposin may contribute to prostate carcinogenesis at its early androgen-dependent or metastatic AI state.

## Background

Androgens, growth factors, neuropeptides, and other trophic agents are involved in normal and neoplastic growth of the prostate. Prosaposin is the intracellular precursor of four lysosomal glycoproteins, saposins A-D, that are involved in lysosomal hydrolysis of sphingolipids. These saposins, through their interaction with glycosphingolipid hydrolases and their substrates, increase lysosomal hydrolytic activities. Saposins and prosaposin are expressed by various cell types and as a secretory protein in body fluids including blood, seminal plasma, seminiferous tubular fluid, and prostatic secretions [1-5]. Prosaposin and its active domain, saposin C, are known for their potent neurotrophic activities and are involved in neuro-embryological development [6,7]. The neurotrophic activity of prosaposin has been attributed to the NH_2_-terminal portion of the saposin C domain of the molecule which is the source for a number of biologically active synthetic peptides such as prosaptides TX14A [4-6]. Prosaptides (i.e., TX14A), saposin C, and prosaposin exert their biological effects by binding to a partially characterized single high-affinity G-protein coupled receptor (GPCR) [6-8]. It has been reported that mice with an inactivated *prosaposin *gene die at 35–40 days of age due to neurological disorders. These mice also develop several abnormalities in their reproductive organs, such as atrophy and involution of the prostate gland and inactivation of MAPK and Akt in the prostate epithelium [9,10]. The spectrum of biological activities of prosaposin or saposin C in cancer biology in general and in prostate cancer has not been specifically addressed.

We have recently reported a higher expression of prosaposin in androgen-independent (AI) prostate cancer cells (PC-3 and DU-145) than in androgen-sensitive (AS) LNCaP or in normal prostate epithelial and stromal cells. In addition, we have found that prosaptide TX14A stimulates prostate cancer cell proliferation, migration, and invasion, activates the Raf-MEK-ERK-Elk-1 signaling cascade of the mitogen-activated protein kinase (MAPK) pathway, and inhibits the growth-inhibitory effects of sodium selenite administered at apoptogenic concentrations [11].

In the present study, we show for the first time that saposin C also functions as a survival factor, activates PI3K/Akt-signaling pathway, and in a cell type-specific manner, modulates the expression of procaspase- and caspase-3, -7, and -9 in prostate cancer cells under serum-starvation stress. We demonstrated that prosaptide TX14A, saposin C, or prosaposin decreased the growth-inhibitory effects, caspase-3/7 enzymatic activity, and apoptotic cell death induced by etoposide. In addition, our data show that saposin C activation of a p42/44 MAPK in prostate cancer cells is not only pertussis toxin-sensitive, but also PI3K/Akt-dependent. Moreover, the PI3K-inhibitor, LY294002, restores the apoptogenic effect of etoposide in prostate cancer cells studied.

We propose that as a survival and anti-apoptotic factor, saposin C or prosaposin may contribute to prostate carcinogenesis or to the development of hormone-refractory prostate cancer.

## Results

### Saposin C acts as a survival factor for prostate cancer cells

**Figure 1:**
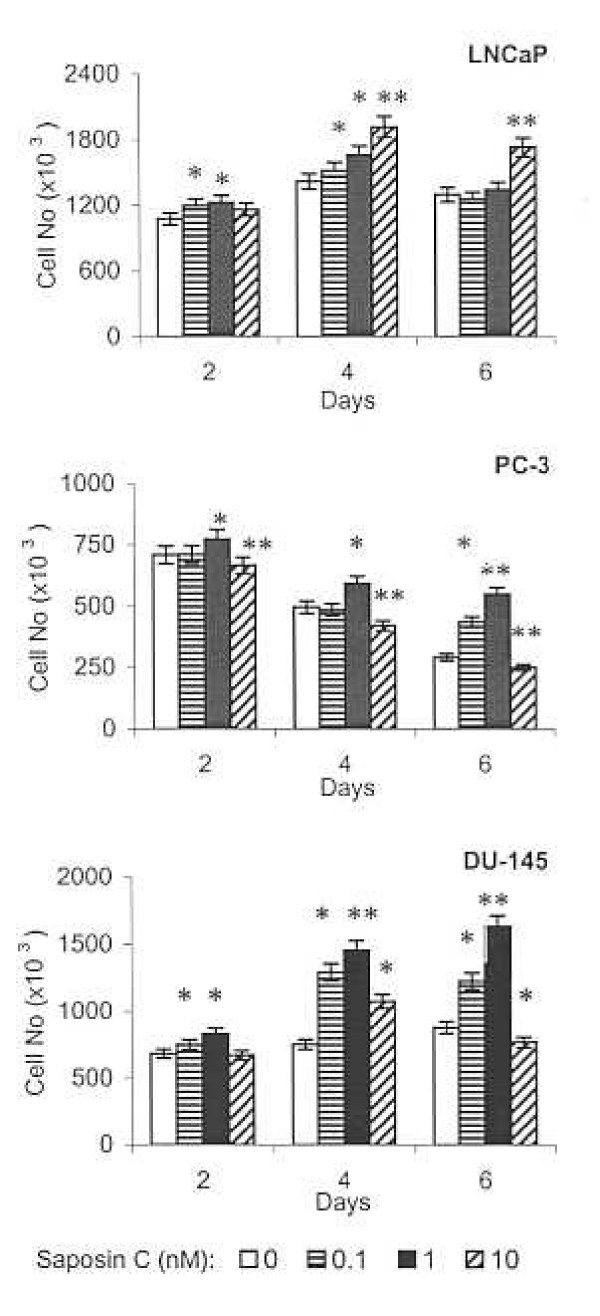
**Saposin C acts as a survival factor for prostate cancer cells. **Cells were cultured in their complete media for 3 days and shifted to their basal (serum-free) media or RPMI-1% FBS (only for LNCaP) in the presence or absence of the indicated concentrations of saposin C for 2, 4, or 6 days. Tissue culture media and saposin C were refreshed every 2 days. At the end of incubation periods, cells were trypsinized and cell number was determined using a hemocytometer and trypan blue exclusion method. PC-3 and DU-145 were used as androgen-independent and LNCaP cells were used as androgen-sensitive prostate cancer cell lines. Data represent the average of three independent experiments in triplicate samples; *bars*, ± SEM. * indicates *P *< 0.05, and ** indicates *P *< 0.01 compared to control. Statistical significance was determined by one-way ANOVA with Bonferroni's corrections.

The effect of saposin C as a survival factor was assessed under serum-starvation stress. Androgen-sensitive (AS) LNCaP cells did not maintain their viability when cultured in serum-free, 0.25% FBS-RPMI, or 0.5% FBS-RPMI media, for more than 36 h. These cells started to detach from tissue culture plates and cell viability was decreased to less than 40% as determined by the trypan blue dye-exclusion method. However, in the presence of 1% FBS, cells remained attached to the tissue culture plate and their growth increased 31% at day 4 and 20% on day 6 as compared with the control values at day 2 (Fig. 1). Saposin C stimulated proliferation of these cells by 13% at day 2, 35% at day 4, and 33% at day 6 compared to the controls. PC-3 cells appeared to be more sensitive to serum deprivation and the number of live cells decreased 30% by day 4 and 60% by day 6 compared to the control values at day 2. However, saposin C (at 1.0 nM) increased cell proliferation by 9% at day 2, 19% at day 4, and 88% at day 6, compared to control plates at the same time period. The growth-response of DU-145 cells was different from PC-3 or LNCaP cells. In the absence of saposin C, the number of live cells increased 10% at day 4 and 29% at day 6 compared to day 2. These cells also demonstrated the highest proliferative response to saposin C at day 4 by 93% (Fig. 1). Taken together, these data indicate that saposin C in a dose-dependent and cell type-specific manner, promotes the survival of the serum-deprived prostate cancer cells.

### Saposin C activates the PI3K/Akt signaling pathway in prostate cancer cells

Several studies have demonstrated that the serine/threonine kinase Akt is a pivotal survival effector for prostate cancer cells and protects them from apoptotic-cell death induction by various types of stresses. Hence, we next evaluated the effect of saposin C on the Akt signaling pathway in cells.

**Figure 2:**
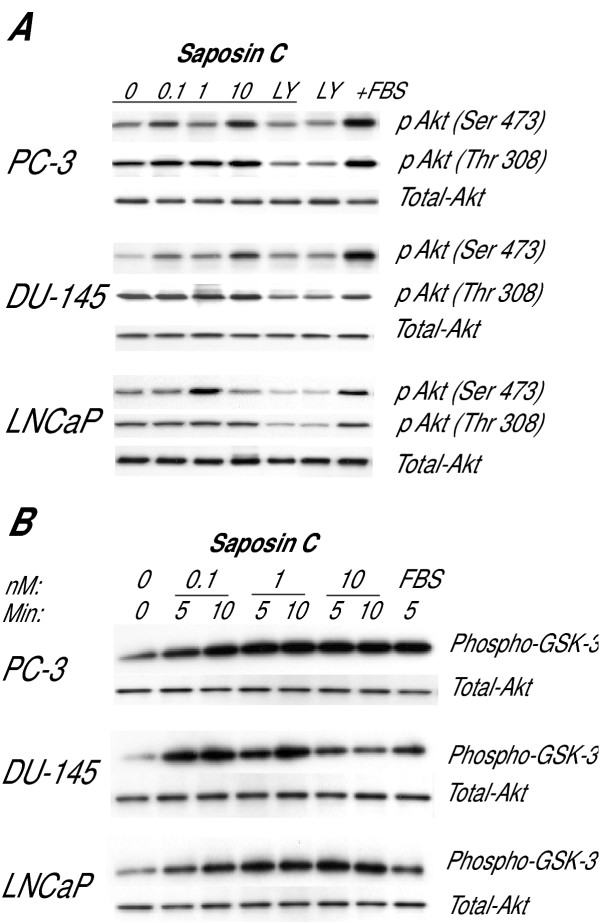
**Saposin C activates Akt signaling pathway in prostate cancer cells. ***A*, cells were cultured up to 70% confluency in their complete media, serum-deprived for 24 h, and treated with 10% FBS or saposin C at 0.1, 1, or 10 nM for 10 min. A representative culture plate was also treated with LY294002 (LY; 50 μM) before treating with saposin C (at 1 nM for LNCaP and 10 nM for PC-3 and DU-145). Fifteen μg protein per sample was subjected to SDS-PAGE under reducing conditions and immunoblotting was carried out using phospho-specific Akt antibodies against serine 473 or threonine 308. *B*, non-radioactive *in vitro *kinase assay was performed to determine the effect of saposin C on Akt kinase activity as described in details in Materials and Methods. Briefly, cells were grown as described above and Akt was selectively immunoprecipitated from 250 μg protein using 20 μl of immobilized Akt 1G1 monoclonal antibody. Immunocomplexes were pelletted and resuspended in kinase buffer in the presence of 200 μM ATP and 1 μg of Akt/PKB substrate-glycogen synthase kinase fusion protein (GSK-3α/β) and incubated for 30 min at 30°C, allowing immunoprecipitated Akt (if activated) to phosphorylate GSK-3. After terminating the kinase reaction, phosphorylated GSK-3 was detected by SDS-PAGE and immunoblotting using phospho-GSK-3α/β antibody. Control loading was evaluated with anti-Akt antibody to determine total Akt-level. Each experiment was performed in duplicate, and the assays were repeated three times.

Direct immunoblotting of serum-starved cells for 24 h showed that saposin C upregulates phosphorylative activity of Akt at serine 473 in androgen-independent (AI) PC-3 and DU-145 cells (Fig. 2A). This response was biphasic. The response of LNCaP cells was distinct and started at 1.0 nM that subsequently returned to a basal level at higher treatment concentrations. Under our experimental conditions, we did not detect any changes in the phosphorylative activity of Akt at threonine 308. Since Akt is a downstream effector of PI3K, we tested the effect of the PI3K-specific inhibitor, LY294002. Pretreatment of cells with LY294002 (50 μM, 3 h) followed by saposin C treatment substantially reduced phosphorylation levels of both serine and threonine residues of Akt in AI- and AD-prostate cancer cells (Fig. 2A).

To determine whether the upregulation of Akt-phosphorylation by saposin C is associated with its kinase activity, *in vitro *kinase assays were performed. After 5 or 10 min exposure of cells to saposin C, activated-Akt induced phosphorylation of glycogen synthetase kinase-3 (GSK-3; a well-characterized Akt substrate) in both AS and AI prostate cancer cells (up to 3-fold compared to basal levels) at concentrations as low as 0.1 nM, was followed by a slight increase at higher concentrations (Fig. 2B). Using purified human milk prosaposin, we observed similar responses (data not shown). The above results indicate that saposin C activates the Akt-signaling pathway in a PI3K-dependent manner in both AS and AI prostate cancer cells.

### Saposin C differentially modulates the expression or activity of caspases and PARP in prostate cancer cells under serum-starvation stress

**Figure 3:**
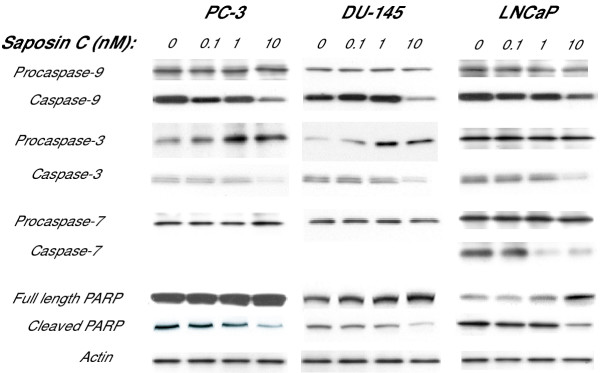
**Effect of saposin C on expression/activity of caspases and PARP under serum-starvation stress. **Cells were cultured routinely up to 60% confluency, washed with PBS, and incubated in their respective serum-free media supplemented with or without saposin C for 48 h. Cell lysates were prepared as described in Materials and Methods and 75 μg of clarified protein samples was subjected to SDS-PAGE under reducing conditions. Western analysis was carried out using monoclonal antibodies against non-cleaved and cleaved caspases-3, -7, and -9 and PARP. For control loading, membranes were probed or reprobed with anti-actin antibody. Each experiment was performed in duplicate, and the assays were repeated three times.

An essential component of the programmed death pathway in many cell types involves proteolytic cleavage of inactive caspases to catalytically active products. We investigated the expression of cleaved (active) and non-cleaved (inactive) forms of the initiator caspase-9, its active downstream effectors (caspases-3 and -7), and poly (ADP-ribose) polymerase (PARP, a nuclear substrate for caspase-3) in the cells after a 48 h serum deprivation period. Caspase-9 is closely coupled to proapoptotic signals and we found that the expression of procaspase-9 was not affected by saposin C; however, we were able to detect a reduction in its cleaved form at 10 nM saposin C in all cells investigated (Fig. 3).

With respect to the effector caspases, we noticed a dramatic dose-dependent increase in the expression of procaspase-3 in the AI PC-3 and DU-145 cell lines. However, we observed a reduction in expression of caspase-3 in both AS and AI cancer cells. Furthermore, our data showed that procaspase-7 expression in these cells was not affected by saposin C and under our experimental conditions we did not detect caspase-7 in AI prostate cancer cells. In LNCaP cells, we found a reduction in the level of caspase-7 at 1 or 10 nM of saposin C (Fig. 3). To further follow the mechanistic response of cells to saposin C in the death cascade, we examined the expression of one of the final death substrates, PARP, and its cleaved product. The intensity of PARP expression was considerably higher in PC-3 and DU-145 cells than in LNCaP cells. Saposin C, in a dose-dependent manner, increased PARP expression and this effect was associated with a parallel dose-dependent reduction of the cleaved (active) PARP levels (Fig. 3). Interestingly, the ratio of PARP: cleaved PARP expression in AI PC-3 and DU-145 cells, either at its basal level or after stimulation with saposin C, was higher than AS LNCaP cells. In general, saposin C induced a cell type-specific (AI versus AS) alteration in the expression level of initiator and effector caspases. This effect suggests a better survival and anti-apoptotic activity of saposin C in AI prostate cancer cells than in AS LNCaP cells.

### Saposin C protects prostate cancer cells from etoposide-induced apoptotic cell death

**Figure 4:**
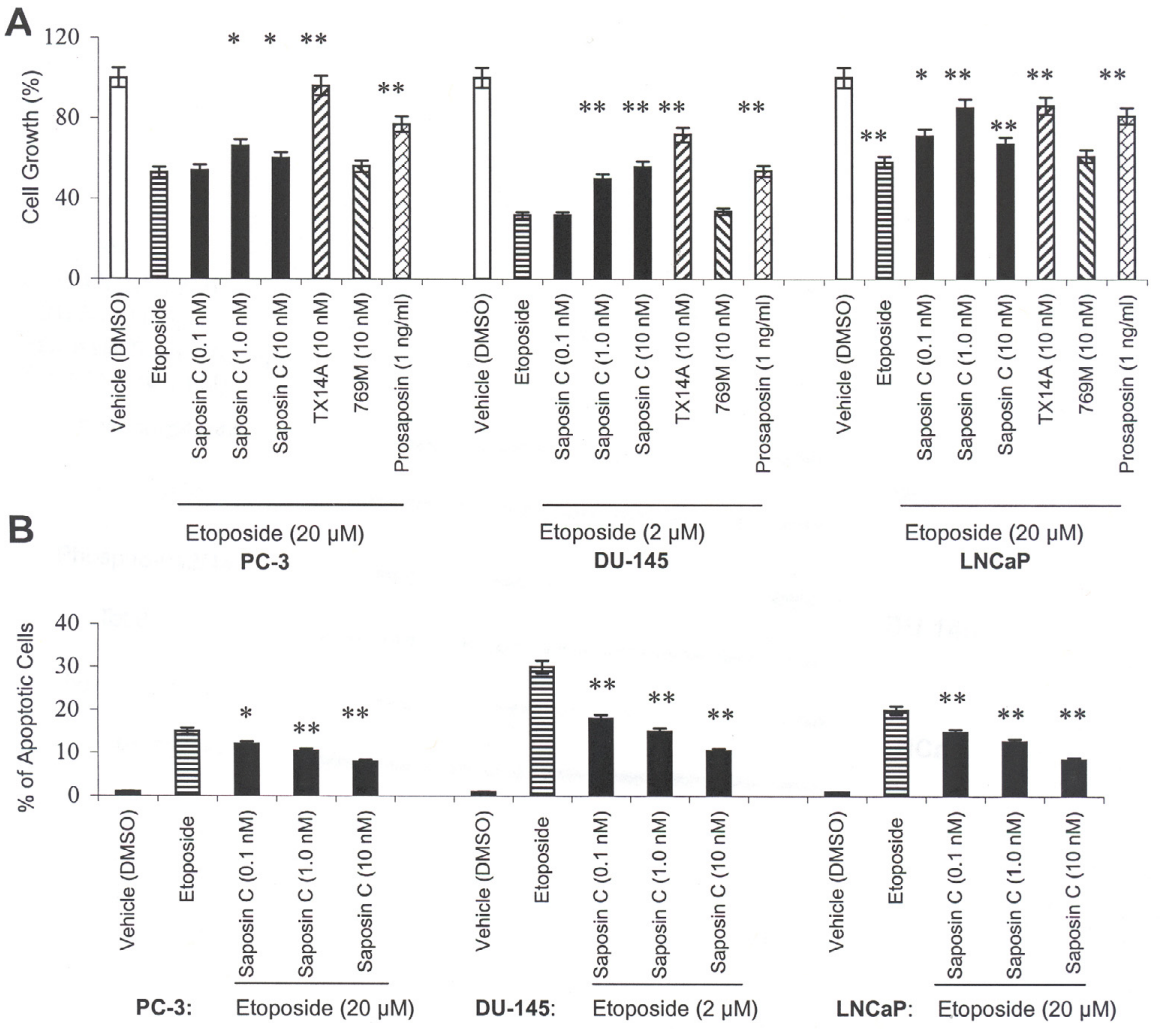
**Saposin C reduces growth inhibitory effect of etoposide and acts as an anti-apoptotic factor for prostate cancer cells. ***A*, cells were seeded at 2000 per well in 96-well plates in their complete culture media for 3 (for PC-3 and DU-145) or 4 days (for LNCaP), treated with vehicle (DMSO), saposin C (0.1, 1, or 10 nM), prosaptide TX14A (10 nM), inactive mutant peptide 769M (10 nM), or prosaposin (1 ng/ml) at the indicated concentrations in the presence or absence of etoposide at the indicated concentrations for 3 days. After this period cell number was determined using MTS assay and cell type-specific OD/cell number calibration curve as described in Materials and Methods. *B*, apoptosis was determined by TUNEL assay. Cells were cultured in multiwell chamber slide up to 40% confluency in their complete culture media, and treated with etoposide in the presence or absence of saposin C (0.1, 1, or 10 nM) for 3 days. Percentage of apoptosis was determined by random selection of 10 microscopic field (at × 200 magnification) and cell count with a hemocytometer. Data expressed at the average of three independent experiments and twelve replicate samples; *bars*, ± SEM. * indicates *P *< 0.05, and ** indicates *P *< 0.01 compared to control (etoposide). Statistical significance of the effect of saposin C on cell growth and apoptosis was evaluated by one-way ANOVA with Bonferroni's corrections. Differences of vehicle (or etoposide)-only treated cells and any other single experimental group of interest (TX14A or prosaposin) was evaluated by Student's *t*-test and statistical significance was set at *P *< 0.05.

Next, we decided to evaluate the effect of an apoptogenic agent, etoposide, on cell growth, apoptosis, and caspase activity in the presence or absence of various effectors. Cells were treated in complete culture media for three days, and then subjected to the MTS assay. Using these experimental conditions, we empirically determined the lowest concentration of etoposide that would lead to the highest growth inhibition. We found that the growth inhibitory effect of etoposide on prostate cancer cells is also cell type-specific. For example, a 20 μM etoposide concentration was sufficient to reduce the cell number to 53% in PC-3 and to 58% in LNCaP cells as compared to their control values. However, DU-145 cells were more sensitive and treating these cells with only 2 μM etoposide led to a 69% reduction in the cell number compared to control values (Fig. 4A). Compared to etoposide-treated cells, saposin C increased cell growth by 13% in PC-3, 24% in DU-145, and 27% in LNCaP cells. Like saposin C, prosaposin reduced etoposide-induced growth inhibition to relatively the same degree. The highest increase in cell number was achieved with synthetic peptide TX14A treatment; however treatment of cells with the mutant 769M peptide showed only a negligible effect (2–5% increase the cell number) (Fig. 4A). These results indicate that TX14A peptide, saposin C, or prosaposin can reduce etopside growth-inhibition on prostate cancer cells.

Using the TUNEL assay and above experimental conditions, we next evaluated the effect of saposin C on the percentage of apoptotic cells after treating cells with etoposide for three days. Apoptotic cells were identified as dense, bright, and punctate, with brownish pigmentation of poly-fragmented nuclei. Among the three cell lines investigated, PC-3 proved to be the most resistant cell line to apoptosis induction by etoposide. Overall, there was a dose-dependent reduction (with a peak effect at 10 nM) of apoptotic cells in the three cell lines investigated. Saposin C decreased apoptotic cells by 47% in PC-3, 89% in DU-145, and 58% in LNCaP cells (Fig. 4B). This result demonstrates the counteracting influence of saposin C and etoposide on apoptosis in prostate cancer cells.

**Figure 5:**
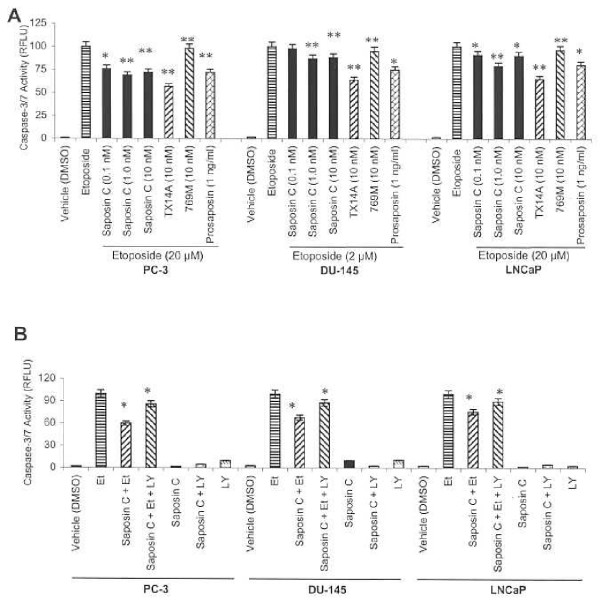
**Effects of saposin C on caspase-3/7 activity (A) and the influence of PI3-kinase inhibitor (B) in etoposide-treated cells. **Cell culture, treatment period, growth and caspase activity was described in Figure 4. Caspase-3/7 activity was determined using the Apo-ONE Homoheneous Caspase-3/7 assay kit based on the cleavage of a profluorescent caspase-3/7 substrate (Z-DEVD-R110) and fluorimetric quantitation was performed at an excitation and emission wavelength of 485+20 and 535+25 nm, respectively. After correction of the fluorimetric reading with the blank (vehicle control), final fluorescent intensity was depicted as an arbitrary endpoint relative fluorescent unit, RFLU. PI3-kinase inhibitor (LY294002; LY) was used at final 1.5 μM concentration and saposin C was added at optimal 1 nM (for PC-3 and LNCaP) or 10 nM (for DU-145) concenration. Etoposide (Et) was added at optimal 20 μM (for PC-3 and LNCaP) or 2 μM (for DU-145). Data expressed are the average of three independent experiments and twelve replicate samples; *bars*, ± SEM. * indicates *P *< 0.05, and ** indicates *P *< 0.01. Statistical significance of the effect of saposin C on cell growth and apoptosis was evaluated by one-way ANOVA with Bonferroni's corrections. Differences of vehicle (or etoposide)-only-treated cells and any other single experimental group of interest (TX14A or prosaposin) was evaluated by Student's *t*-test and statistical significance was set at *P *< 0.05.

We also employed a sensitive fluorometric assay to measure caspase-3/7 (based on DEVDase) activity using the experimental conditions described above. Saposin C, at 1 nM concentration, demonstrated the highest reduction in caspase-3/7 activity in AS LNCaP (21%) and in AI PC-3 (35%) and DU-145 cells (30%) (Fig. 5A). Prosaposin-treated cells also demonstrated a similar effect. TX14A peptide not only decreased the growth-inhibitory effect of etoposide (data not shown), but also proved to be a potent anti-apoptotic peptide, reducing caspase-3/7 activity by 43% in PC-3, 36% in DU-145, and 30% in LNCaP cells. However, the control (inactive mutant) peptide's (769M) effect was minimal (with a 2–5% reduction) (Fig. 5A). These results clearly indicate that the anti-apoptotic activity of saposin C is at least partially associated with modulation of caspase-activity.

### The PI3K/Akt inhibitor restores apoptogenic activity of etoposide in saposin C treated cells

To determine whether or not saposin C anti-etoposide apoptotic activity is PI3-kinase dependent, the effect of PI3K/Akt inhibitor (LY294002) on caspase-3/7 activity in the cells was examined in the presence or absence of saposin C ± etoposide. Through our initial studies, using trypan blue exclusion and MTS assays, we found 1.5 μM of LY294002 was a non-toxic and tolerable dosage for experimental period (3 days).

Compared to DMSO-treated cells, LY294002 slightly increased (up to 10%) the caspase-3/7 enzymatic activity in PC-3 and DU-145 and showed almost no change in LNCaP (Fig. 5B). As described above, saposin C significantly decreased induction of caspase-3/7 activity by etoposide. Saposin C also reduced the induction of casapases activity by LY294002 under our experimental conditions. Addition of LY294002 to the cells treated with saposin C and etoposide increased caspase-3/7 enzymatic activity, but to a level below than etoposide-only treated cells (Fig. 5B). These results indicate that antiapoptotic activity of saposin C and its effect on caspase activity is at least partially mediated via the PI3K/Akt signaling pathway.

### Saposin C activation of MAPK is pertussis toxin-sensitive and PI3K/Akt-dependent

In neuro-glial derived cells, neurotrophic activity, cell-death protection and the activation of MAPK by prosaptides (i.e., TX14A), saposin C, or prosaposin are mediated by their binding to a pertussis toxin-sensitive GPCR [6,7,12,13]. Our previous data demonstrated that prostate cancer cells were differentially responsive to the TX14A peptide in a number of biofunctional assays [11]. Our current results indicate the presence of a sensitive and /or responsive receptor-ligand interaction that could be accountable for the subsequent activation of downstream signaling effectors in MAPK- and Akt-signal transduction pathways. In addition, there is also emerging data indicating that signaling proteins such as PI3K and Akt can also activate MAPK pathways [14-16].

**Figure 6:**
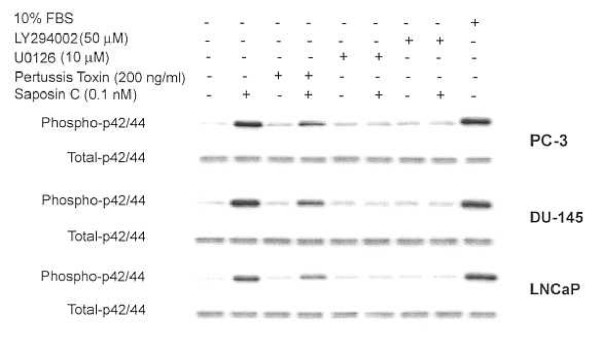
**Saposin C activation of MAPK pathway is pertussis toxin-sensitive and PI3K/Akt-dependent. **Cells were cultured up to 60% confluency in their maintenance media, washed with PBS, serum-deprived for 20 h, and then fresh basal culture media was added for an additional 4 h in the presence or absence of LY294002 (50 μM, 3 h), Wortmannin (10 μM, 15 min), U0126 (10 μM, 1.5 h), or pertussis toxin (200 ng/ml, 4 h). After pretreatment, saposin C (0.1 nM) was added directly to the cells and incubated for 5 min at 37°C. Cell lysates were prepared and 10 μg protein per sample was subjected to SDS-PAGE and immunoblotting using phospho-specific p42/44 MAPK antibody. For control loading, membranes were also probed or reprobed with p42/44 antibody to detect total p42/44 MAPK. Parallel tissue culture plates, treated in the same manner, were also tested for cell viability by trypan blue dye-exclusion assay. FBS at 10% final concentration was used as a positive control. Each experiment was performed in duplicate, and the assays were repeated three times.

We have previously demonstrated MAPK-activation by the TX14A peptide derived from a trophic sequence of saposin C [11]. To examine the involvement of GPCR in saposin C-activation of the Akt-signaling pathway as well as the possibility of MAPK and Akt cross-signaling initiated by saposin C, we evaluated the effect of saposin C on p42/44 MAP kinase activation in prostate cancer cells in the presence or absence of various inhibitors. Treatment of cells with saposin C increased the phosphorylative activity of p42/44 MAPK, which was substantially inhibited by pretreating cells with the specific MEK inhibitor (U0126; Fig. 6). We used 10% FBS treatment as an external positive control for induction of p42/44 activity in the cells. Saposin C treatment of cells pretreated with PT showed a modest (in LNCaP cells) to strong reduction (in AI prostate cancer cells) in the level of phospho-p42/44 MAPK. This result clearly indicates a cell type-specific PT-sensitivity and/or the potential involvement of one or more G-proteins in saposin C-activation of MAPK pathway in the cells.

The intensity of reduction of p42/44 activity and its cell type-specific pattern was very similar to PT plus saposin C- or PT-treated values. Interestingly, we observed a more profound reduction in p42/44 phosphorylative activity in cells pretreated with LY294002 and then treated with saposin C. To rule out the potential cytotoxic effect of the inhibitors, viability of cells was also determined by trypan blue dye-exclusion. We observed essentially similar results using the other structurally and mechanistically different PI3-kinase inhibitor (wortmannin). This experiment revealed that at the end of the pre-treatment incubation period, cell viability was equal to or more than 95%. These results indicate that MAPK activation by saposin C is at least partially mediated by saposin C-regulated PI3K/Akt pathways in prostate cancer cells. This result also provides additional proof for simultaneous activation of multiple (inter-related) signal transduction pathways by saposin C.

## Discussion

Induction of apoptosis by androgen-ablation therapy significantly reduces androgen-dependent (AD) prostate cancer cells, but fails to cure the majority of patients due to the presence of apoptosis-resistant cancer cells that are androgen-independent [17]. It is likely that the development of these cells is an adaptive response to hormonal therapy rather the overgrowth of resistant cells. In-depth understanding of apoptotic phenomena, identification of its intracellular components, and characterization of its extracellular effectors as inducers or inhibitors may contribute to therapeutic approaches for prostate cancer.

The *prosaposin *knock-out mouse model has revealed a number of interesting findings specifically in the male reproductive organs. Among these are atrophy of the prostate gland, epididymis, and seminal vesicles. Microscopic evaluation of affected tissues also shows undifferentiated phenotypes in prostate ventral and dorsal lobules and atrophy of the tubuloalveolar glands and their epithelial cell lining [9]. These data are suggestive of a primary role for prosaposin in development of the prostate gland.

In a variety of neuro-glial derived cells, synthetic peptides encompassing a trophic sequence of saposin C and/or prosaposin have been found to induce growth, survival, and/or differentiation, or to prevent apoptotic cell-death *in vitro *and *in vivo *[11,18-20]. For example, prosaptide-TX14A and prosaposin, in a dose- and time-dependent manner reduced apoptotic cell-death induction of primary Schwann cells cultured in low serum concentrations, and PI3K inhibitors (wortmannin or LY294002) blocked their anti-apoptotic effects [21].

Here, we found that under prolonged serum starvation (2 to 6 days), although the responses of androgen-sensitive (AS) LNCaP and AI PC-3 and DU-145 prostate cancer cells were different from each other, saposin C in a dose- and time-dependent manner, proved to increase proliferation and survival of both cell types (Fig. 1). Normal prostatic epithelial cells neither tolerated the basal medium nor responded to saposin C (data not shown). Like many other cancer cells, withdrawal of mitogens, growth factors, and other trophic factors by serum-starvation, serves as a potent stimulus and driving force activating different survival mechanisms that eventually lead to apoptotic cell-death in AD- and Al-prostate cancer cells [22,23]. Since the PI3-Kinase/Akt signaling pathway is known as a central cell-survival mechanism and an important mediator of survival signals driven by growth or trophic factors, we were also interested in examining the level of PI3K/Akt activity during serum starvation. Interestingly, we found that saposin C upregulated Akt-p473^Ser ^phosphorylative activity in the prostate cancer cells under investigation. This effect was substantially inhibited by LY294002, an inhibitor of the upstream Akt effector, PI3K (Fig. 2A). In addition, using an *in vitro *kinase assay, we proved that saposin C induction of PI3K/Akt activity in cells was associated with increased phosphorylation of GSK3α/β, as a downstream key target of the Akt kinase (Fig. 2B). Unlike Akt-p473^Ser^, our study showed a considerably higher level of constitutively activated Akt-p308^Thr ^in all cells and remained unaffected by saposin C. Other studies have also supported a central role for the PI3K/Akt pathway as a dominant growth factor-induced survival pathway for prostate cancer cells [14,16]. Constitutive activation of the PI3K/Akt survival pathway has been described as a mechanism that enables endocrine-related breast, lung, and prostate cancer to become refractory to cytotoxic therapy [24-27]. Interestingly, immunohistochemical analyses have demonstrated a direct correlation between Akt phosphorylation and the Gleason's score in prostate cancer [28]. Our results provide evidence that implicate the involvement of PI3K/Akt activity in saposin C (growth factor)-induced survival of prostate cancer cells.

With respect to prosaposin, immunohistochemical staining of the involuted and atrophied prostate tissues from homozygous knock-out mice also showed inactivation of the MAPK and Akt signaling pathways [9,10]. Apoptotic-death signal transduction pathways are not limited to PI3K/Akt and may involve multiple redundant physiological pathways. Several studies have shown the involvement of caspases in the apoptosis of the prostate gland in normal development or malignant conditions [29,30]. For example, immunohistochemical evaluation in castrated mice and rats showed the presence of (activated) caspases in prostate and a correlation between caspase-3 expression and the Gleason's grade of tumors [29]. *In vitro *studies also revealed that caspase-inhibitory mechanisms might be involved in metastasis of prostate cancer cells [15]. We found that saposin C, in a dose-dependent manner, increased procaspase-3 and PARP levels and decreased the cleaved form of caspase-9 and -3 and PARP (a caspase-3 substrate) in both AS and AI prostate cancer cells. PARP cleavage has been recognized as a sensitive marker of caspase-mediated apoptosis and its cleavage paralyzes the enzyme's ability to repair DNA strand breaks. Therefore, reduction of the PARP cleavage is a strong indicator for anti-apoptotic activity of saposin C. Although procaspase-7 expression was not affected in any of the cells investigated, its active form was reduced only in LNCaP cells and was not detected in AI prostate cancer cells (Fig. 3). This special pattern for alteration in the level of procaspase-3, its cleaved form, and PARP was coincident with saposin C-induced cell survival under serum-deprivation culture condition (Fig. 1). Such divergent regulation of caspase-3 and PARP has rarely been reported in prostate cancer cells [31]. However, it has been demonstrated frequently in the nervous system and therefore might represent a unique characteristic of prosaposin or saposin C as a neurotrophic molecule [32].

Next, we exposed cells to a universal apoptogenic agent, etoposide, and found that prosaposin or its active derivatives (saposin C or TX14A peptide), were able to decrease the growth-inhibitory effect of etoposide-treated prostate cancer cells (Fig. 4). TUNEL assay, as a direct measure of apoptotic death showed a dose-dependent reduction in the percentage of apoptotic cells by saposin C (Fig. 4B). Under similar experimental conditions, we also showed that saposin C, prosaptide TX14A, or prosaposin reduce caspase-3/7 activity in cells treated with etoposide. This effect could be counteracted by administration of a PI3-kinase inhibitor (LY294002) (Fig. 5A and 5B). These data are a clear indication that saposin C-inhibition of the apoptogenic activity of etoposide is at least partially dependent on the upstream Akt effector, PI3K (Fig. 5B). Together, the above findings suggest that the two closely inter-connected cell survival/apoptotic pathways (PI3K/Akt and caspases) activated by saposin C or prosaposin might potentially synergize and provide a growth and survival advantage to both AD- and AI-prostate cancer cells.

Induction of mitogenic, survival, and anti-apoptotic signals in physiological and pathological conditions may begin from a wide array of extracellular stimuli and receptors, including receptor tyrosine kinases (RTKs) and G-protein coupled receptors (GPCRs). From a historical point of view, considerable attention has been given to the role of RTKs and their cognate polypeptide ligands in prostate cancer. However, accumulating evidence support the involvement of lysophosphatidic acids, neurotransmitters, and neuropeptides such as bombesin and neurotensin through GPCR signaling in the initiation or progression of prostate cancer [32,33]. GPCRs also use pathways that are very similar to those utilized by RTKs to activate survival and anti-apoptotic signaling pathways such as the prototypic Raf-MEK-MAPK and PI3K/Akt signal transduction pathways and caspase cascades [32,34-37].

Here, we showed that saposin C, in a pertussis toxin-sensitive manner, activated p42/44 MAPK (Fig. 6). Our study demonstrated the involvement of GPCR as a responsible receptor system interacting with saposin C and therefore activating the subsequent signaling pathways. Due to the considerable importance of activation of MAPK signal transduction activation by saposin C-GPCR and activation of the Akt signaling pathways, we tested whether inhibition of PI3K/Akt could affect saposin C-induced p42/44 MAPK activation. Pretreatment of cells with LY294002 inhibited saposin C activation of p42/44 MAPK (Fig. 6). These data not only indicate cross-communication between MAPK- and PI3K/Akt-signaling pathways, but also might suggest that simultaneous activation of the two important signal transduction pathways by saposin C provide a potent cell survival and apoptotic-death protection program for prostate cancer cells.

## Conclusion

Our data for the first time show that by activation of multiple inter-related signaling pathways (PI3K/Akt and MAPK) and cell type-specific modulation of expression or activity of caspases, saposin C and/or its precursor (prosaposin) serve as a survival and anti-apoptotic factor for both AS- and AI-prostate cancer cells. Elucidation of such intricate mechanisms could potentially provide a therapeutic option that combines cytotoxic therapy and inhibition of survival/anti-apoptotic signals for AI metastatic prostate cancer. Finally, our observations provide novel insights into the diversity of biological activities of prosaposin in prostate cancer cells.

## Methods

### Cell lines

Androgen-independent (PC-3, DU-145) and -sensitive (LNCaP) prostate cancer cell lines were obtained from the American Type Culture Collection (Manassas, VA) and grown in defined media (PC-3 and DU-145 in DMEM-10% FBS and LNCaP in RPMI-1640-10% FBS supplemented with 1 mM sodium pyruvate, 10 mM HEPES). Purified recombinant human saposin C and prurified human milk-prosaposin were characterized and provided by Dr. K. Sandhoff (University of Bonn, Germany) and Dr. M. Hiraiwa (University of California, San Diego), respectively.

### Cell survival assays

Cells were initially grown in 100 mm plates in their respective culture media for 3 days, and after washing with PBS were incubated in serum-free DMEM (PC-3 and DU-145) or RPMI-1% FBS (LNCaP) in the presence or absence of saposin C (at 0.1, 1, or 10 nM) for 2, 4, or 6 days. Saposin C and culture media were replaced every 2 days. At the end of the incubation periods, cells were trypsinized and cell number was determined using a hemocytometer and the trypan blue exclusion method.

### Western analysis

Protein expression analysis was performed according to standard procedures [38]. Briefly, the cell extract was prepared by washing cell monolayers with cold-PBS, lysing the cells on ice for 15 min with lysis buffer (20 mM PIPES [pH 7.4], 150 mM NaCl, 1 mM EGTA, 1% Triton X-100, 1.5 mM MgC1_2_) supplemented with a protease inhibitor cocktail (Roche Diagnostic, Inc., Indianapolis, IN) and 1 mM sodium orthovandate, plus sodium dodecyl sulfate (SDS) at a final concentration of 0.1%. The lysates were then centrifuged (15 min, 4°C, 16,000 × g), aliquoted, and stored at -70°C until use. Protein concentration was determined by BCA assay (PIERCE, Rockford, IL). Each experiment was repeated at least two times. For western analysis, membranes were blocked with 5% BSA in the rinse buffer (150 mM NaCl, 20 mM Tris, 0.1% Tween 20) for 1 h, washed in rinse buffer for 10 min, and then incubated with the respective primary antibody at the indicated concentrations (see below). The membranes were then washed and incubated with the appropriate horseradish peroxidase-conjugated secondary antibody (1:1000 dilution; Santa Cruz Biotechnology, Santa Cruz, CA) for 1 h at room temperature, washed for 10 min and four more cycles of 5 min, and treated with an enhanced chemiluminescence (ECL) detection system (Amersham, Piscataway, NJ). In some cases, when the signal was very weak or undetectable, we used ECL-plus (Amersham).

#### (i) Effect of PI3K/Akt and MEK-inhibitors, or Pertussis Toxin on Saposin C Activation of p42/44 MAPK

Cells were grown in their respective complete culture media for 2–3 days (up to 60% confluency), washed with PBS, incubated in their serum-free (basal) media for 20 h, and then fresh basal media was added to all plates for an additional 4 h. Various inhibitors [LY294002 (50 μM, 3 h), Wortmannin (10 μM, 15 min), U0126 (10 μM, 1.5 h), and Pertussis toxin (200 ng/ml, 4 h)] were added to the culture medium, just before treating cells with saposin C (at 0.1 nM, 5 min). We used 10% FBS as a positive control. Cells were lysed and 10 μg of clarified protein samples was subjected to SDS-PAGE under reducing conditions. Phospho-specific p42/44 antibody (1:1000; Cell signaling Technologies, Bedford, MA) was used as the primary antibody and as a loading control. Filters were also probed or reprobed with anti-p42/44 antibody. Additional tissue culture plates that had been treated with or without inhibitors were also tested for cell viability by trypan blue dye-exclusion assay.

#### (ii) Saposin C and PI3K/Akt signaling pathway

Cells were cultured up to 70% confluency in their complete media and after washing with PBS, they were serum-starved for 24 h, and then treated with 10% FBS or saposin C at 0.1, 1 or 10 nM for 10 min. A representative tissue culture plate was also pretreated with the PI3K-inhibitor (LY294002, 50 μM for 3 h) before treating cells with saposin C (at 1 nM for LNCaP and at 10 nM for PC-3 and DU-145 cells). After preparation of the cell lysate, 15 μg of protein per sample was subjected to SDS-PAGE under reducing conditions. Immunoblotting was performed using phospho-specific Akt antibodies against serine 473 or threonine 308 (Cell Signaling Technology). A loading control was evaluated with anti-Akt antibody. Each experiment was performed in duplicate, and the assays were repeated three times.

### Immunoprecipitation and in vitro Akt kinase activity assay

A non-radioactive Akt kinase assay kit (Cell Signaling Technologies) was used to determine whether saposin C treatment of cells under serum-starvation stress would lead to Akt-activation. For Akt-kinase assays, cells were grown up to 70% confluency in their maintenance media, serum-starved for 24 h, and then treated for 5 or 10 min in the presence or absence of saposin C at 0.1, 1, or 10 nM. Cells were washed once with ice-cold PBS and harvested under nondenaturing conditions using 1X ice-cold cell lysis buffer (from the Kit) supplemented with 1 mM phenylmethylsulfonyl fluoride (PMSF) on ice for 5 (or 10) minutes. Akt was selectively immunoprecipitated from 250 μg protein (whole cell lysates) using 20 μl of immobilized Akt 1G1 monoclonal antibody, and then incubated with gentle rotation for 4 h at 4°C. Samples were then centrifuged briefly (30 sec, 2000 × g) and pellets were washed twice with 1X lysis buffer and once with 1X kinase buffer. Immunocomplexes (pellets) were resuspended in 40 μl 1X kinase buffer [composed of 25 mM Tris (pH 7.5), 5 mM β-glycerolphosphate, 2 mM DTT, 0.1 mM Na_3_VO_4_, and 10 mM MgC1_2 _supplemented with 200 μM ATP and 1 μg glycogen synthase kinase-3 [GSK-3; a well characterized Akt/PKB substrate] of fusion protein (GSK-3α/β) and incubated 30 minutes at 30°C, allowing immunoprecipitated Akt (if activated) to phosphorylate GSK-3. The kinase reaction was terminated by adding 20 μl of 3 × SDS sample buffer. Phosphorylated GSK-3 was then detected by western analysis using phospho-GSK-3α/β (for Ser 21 of GSK-3α and ser 9 of GSK-3β) antibody. The above in vitro kinase assay is based on the fact that phosphorylated-Akt (active) regulates GSK-3α/β kinase activity via phosphorylation at ser 21/9. For control loading, 10 μg protein per sample from the same whole cell lysates were subjected to western analysis using monoclonal anti-Akt antibody or actin. Each experiment was performed in duplicate, and the assays were repeated three times.

### Apoptosis assays

#### (i) Effect of saposin C on expression of caspases by western analysis

Cells were cultured up to 60% confluency in their complete culture media. After washing with PBS, they were incubated with their respective serum-free media in the presence or absence of saposin C at 0.1, 1, or 10 nM for 48 h; whole cell lysates were prepared as described above. Clarified protein samples (75 μg) were subjected to SDS-PAGE under reducing conditions. Western analyses were carried out using monoclonal antibodies against non-cleaved and cleaved caspases-3, -7, and -9 and poly (ADP-ribose) polymerase (PARP) provided in an Apoptosis Sampler Kit (Cell Signaling Technology). Each experiment was performed in duplicate, and the assays were repeated three times.

#### (ii) Fluorometric measurement of caspase-3/7 activity in cells treated with etoposide

We next examined the effect of an apoptogenic agent, etoposide, on cell growth and caspase activity in the presence or absence of saposin C, prosaposin, prosaptide TX14A, or an analogous inactive mutant peptide (769M; 4). Cells were seeded at 1,000 per well in 96-well plates in their complete culture medium for 3 to 4 days. After this period, cells were treated in complete culture media for 3 days with etoposide (2, 20, or 200 μM) to find the lowest concentration that led to the highest growth inhibition, as measured by MTS assay (described above). Using the optimal cell type-specific etoposide concentration (20 μM for PC-3 and LNCaP and 2 μM for DU-145), cells were treated with the vehicle (DMSO), saposin C (0.1, 1, 10 nM), TX14A (10 nM), mutant peptide (769M, 10 nM), or prosaposin (at 1 ng/ml). Using the cell type-specific OD/cell number calibration curve as obtained by MTS assay (Promega, WI), cell number per well was determined for the above treatment conditions.

Parallel tissue culture plates were also used to determine caspase-3/7 activity using the Apo-ONE™ Homogeneous Caspase-3/7 assay (Promega, WI). This assay provides a homogeneous Caspase-3/7 reagent (Promega, Technical Bulletin-TB295) which performs a dual function, by rapidly and efficiently permeabilizing the cultured cells and at the same time exposing the intracellular space to the profluorescent caspase-3/7 substrate, rhodamine 110 (Z-DEVD-R110). After cleavage and removal of the DEVD peptides by caspase-3/7 activity, the fluorescence in each well was quantitated at an excitation wavelength of 485 + 20 nM and an emission wavelength of 535 + 25 nM and after correction based on blank control (DMSO-treated cells at a concentration equal to what used for dissolving etoposide) or the homogeneous caspase-3/7 reagent. Final fluorescent intensity was depicted as endpoint relative fluorescent unit, RFLU.

Using similar experimental conditions but as an independent study, the effect of LY294002 (a PI3-kinase inhibitor) was also evaluated on growth and caspase-3/7 activity of cells treated with saposin C ± etoposide. After initial studies to find the optimal concentration, we used a non-toxic tolerable dosage of 1.5 μM for LY294002. In addition, we chose the most effective (optimal) concentration of etoposide (20 μM for PC-3 and LNCaP and 2 μM for DU-145) and saposin C (1.0 nM for PC-3 and LNCaP and 10 nM for DU-145) for this study.

#### (iii) Terminal deoxynucleotide transferase-mediated nick end-labeling (TUNEL)

Cells were cultured in multiwell chamber slides and treated with etoposide in the presence or absence of saposin C at 0.1, 1, or 10 nM as indicated above. *In situ *determination of apoptosis by Terminal dUTP nick-end labeling (TUNEL) was performed using an ApopTag Peroxidase *In Situ *kit as recommended by the manufacturer (Chemicon International, Temecula, CA). The ApopTag Kit detects single- and double-stranded DNA breaks associated with apoptosis. Drug-induced DNA damage is not identified by the TUNEL assay unless it is coupled with the apoptotic response. Briefly, at the end of the incubation period, cells were fixed in 1% paraformaldehyde in PBS, pH 7.4 for 10 min at room temperature, washed with PBS-twice, and permeabilized in pre-cooled ethanol: acetic acid (2:1) for 5 min at -20°C. After washing twice in PBS, 5 min each time, endogenous peroxidase activity in the cells was quenched in 3% H_2_O_2 _in PBS for 5 min at room temperature, incubated with terminal deoxynucleotidyl transferase (TdT enzyme) and then with peroxidase-conjugated anti-digoxigenin antibody. Nuclear staining of the apoptotic cells was detected by 3',3'-diaminobenzidine tetrahydrochloride dihydrate substrate, as recommended by the manufacturer. Cells were then counterstained in 0.5% (w/v) methyl green and slides were mounted under a glass coverslip in permount mounting medium.

For control staining, the enzyme incubation step was deleted. Microscopic examination of cells was carried out using a phase contrast microscope. Cells were counted by choosing ten random fields and the percentages of apoptotic cells were determined. Apoptosis was indicated by the presence of apoptotic bodies, exhibiting brightly labeled punctuated nuclei.

### Statistical analyses

For cell survival and other quantitative data, a one-way analysis of variance (ANOVA) was employed to evaluate the influence of one variable on multiple independent groups. Bonferroni's corrections were also applied whenever a significant group effect was observed. To compare a control group with a single experimental group of interest, we used the Student's t-test. For cell survival studies, each treatment concentration was examined three times and in triplicate samples. The effect of saposin C or other effectors in the presence of etoposide on cell growth, apoptosis, or caspase-3/7 activity was studied in twelve replicates and repeated three times. Statistical significance was set at *p *< 0.05 or 0.01. Statistical analyses were performed using GraphPad Prism version 3.00 for Windows (GraphPad Software, San Diego, CA).

## Authors' contributions

Author 1 (T-JL) carried out experiments described in figure 4 and 5B. Author 2 and 3 (OS and RL) reviewed the manuscript and provided valuable comments in different sections of the manuscript. Author 4 (SK) conceived the study, designed all the experiments, performed experiments described in figures 1, 2, 3, 5A, and 6, carried out the statistical analysis, and drafted the paper and wrote the final version of the manuscript. All authors read and approved the final manuscript.
